# Evaluation of a Social Media Campaign in Saskatchewan to Promote Healthy Eating During the COVID-19 Pandemic: Social Media Analysis and Qualitative Interview Study

**DOI:** 10.2196/27448

**Published:** 2021-07-21

**Authors:** Jordyn L Grantham, Carrie L Verishagen, Susan J Whiting, Carol J Henry, Jessica R L Lieffers

**Affiliations:** 1 College of Pharmacy and Nutrition University of Saskatchewan Saskatoon, SK Canada

**Keywords:** COVID-19, diet, healthy, nutrition, health promotion, social media, dietitian, Saskatchewan

## Abstract

**Background:**

The beginning of the COVID-19 pandemic presented many sudden challenges regarding food, including grocery shopping changes (eg, reduced store hours, capacity restrictions, and empty store shelves due to food hoarding), restaurant closures, the need to cook more at home, and closures of food access programs. Eat Well Saskatchewan (EWS) implemented a 16-week social media campaign, #eatwellcovid19, led by a dietitian and nutrition student that focused on sharing stories submitted by the Saskatchewan public about how they were eating healthy during the COVID-19 pandemic.

**Objective:**

The goal of this study was to describe the implementation of the #eatwellcovid19 social media campaign and the results from the evaluation of the campaign, which included campaign performance using social media metrics and experiences and perspectives of campaign followers.

**Methods:**

Residents of Saskatchewan, Canada, were invited to submit personal stories and experiences to EWS about how they were eating healthy during the COVID-19 pandemic from April to August 2020. Each week, one to three stories were featured on EWS social media platforms—Facebook, Instagram, and Twitter—along with evidence-based nutrition information to help residents become more resilient to challenges related to food and nutrition experienced during the COVID-19 pandemic. Individuals who submitted stories were entered into a weekly draw for a Can $100 grocery gift card. Social media metrics and semistructured qualitative interviews of campaign followers were used to evaluate the #eatwellcovid19 campaign.

**Results:**

In total, 75 stories were submitted by 74 individuals on a variety of topics (eg, grocery shopping, traditional skills, and gardening), and 42 stories were featured on social media. EWS shared 194 #eatwellcovid19 posts across social media platforms (Facebook: n=100; Instagram: n=55; and Twitter: n=39). On Facebook, #eatawellcovid19 reached 100,571 followers and left 128,818 impressions, resulting in 9575 engagements. On Instagram, the campaign reached 11,310 followers, made 14,145 impressions, and received 823 likes and 15 comments. On Twitter, #eatwellcovid19 made 15,199 impressions and received 424 engagements. Featured story submission posts had the best engagement on Facebook and the most likes and comments on Instagram. The EWS social media pages reported increases in their following during the campaign (Instagram: +30%; Facebook: +14%; and Twitter: +12%). Results from the interviews revealed that there were two types of campaign followers: those who appreciated hearing the stories submitted by followers, as it helped them to feel connected to the community during social isolation, and those who appreciated the evidence-based information.

**Conclusions:**

Numerous stories were submitted to the #eatwellcovid19 social media campaign on various topics. On Instagram and Facebook, posts that featured these stories had the highest engagement. During this campaign, EWS’s social media following increased by more than 10% on each platform. The approach used for the #eatwellcovid19 campaign could be considered by others looking to develop health promotion campaigns.

## Introduction

On March 11, 2020, the World Health Organization declared the disease caused by the SARS-CoV-2 coronavirus, COVID-19, a pandemic [1]. COVID-19 is a respiratory disease with many symptoms, including fever, dry cough, and fatigue [2]. Respiratory droplets are the main source of disease transmission, and individuals are most likely to become infected when they are in close contact with others who have COVID-19 [3]. Although approximately 80% of people who develop symptoms from COVID-19 recover without hospitalization, this disease is particularly concerning as about 15% of people who have symptoms of COVID-19 require hospitalization, and about 5% require intensive care treatment [2]. Containment measures, including restrictions on travel; closing nonessential businesses, services, and schools; requiring and encouraging people to work from home; social distancing; handwashing; and use of face masks, have been put in place in attempts to contain the virus [4-7].

The COVID-19 pandemic presented many challenges related to food, nutrition, and eating habits. Food access and disruptions to food environments were a major concern at the beginning of the pandemic. The social distancing measures also caused abrupt changes in food sourcing patterns [8]. One of the first impacts was a stage of food hoarding and panic shopping; in Canada, grocery store sales increased by 38% the week of March 11, 2020, compared to 2019, with the sale of dried goods, frozen foods, and shelf-stable foods having greatly increased [9]. Residents also experienced price fluctuations that limited purchasing power, and there were increases in demand for online grocery shopping [10]. Food hoarding and panic buying behaviors that took place early in the pandemic reflected consumers’ concerns regarding decreased food availability and uncertainty in the stability of the supply chain [11]. Grocery shopping habits and patterns were disrupted as consumers attempted to comply with the new public health recommendations, which included advising that a single member of the household shop for food biweekly [10,12,13]. In addition, numerous social distancing measures were put in place at food stores, which further complicated grocery shopping [10]. Purchasing food to last for 2 weeks was overwhelming, especially for those with reduced purchasing power, impacting the types of meals families could prepare and requiring increased planning, a difficult task when food availability is uncertain and time is limited in food stores [10,13]. Prior to the pandemic, there were also concerns surrounding where the public obtains nutrition information [14,15], and the pandemic may have created more concern around this. In addition, restaurants and smaller food retailers were suddenly closed or had to take time to shift service styles, disrupting the food environment further [16,17]. There were also concerns about food safety (eg, handling of produce at the grocery store and transmission via surface contamination of frozen foods) [18-21]. Vulnerable populations, those already food insecure, and those experiencing employment instability were most affected by the abrupt disruptions to food environments and food access issues and were more likely to experience food insecurity as a result, but these food access issues impacted people across socioeconomic levels [22-25].

The abrupt changes to the daily routines due to COVID-19 had impacts on food preparation and eating habits; studies have reported both positive and negative impacts. For example, many people began working from home or participating in online learning, and this led to increased time spent cooking, more time eating together as a family, reduced take-out food consumption, increased overall food consumption, and increased calories from snack food consumption [26]. More time spent at home also meant parents were fielding increased snack requests from children and dealing with the temporary loss of school-provided meals [22,26,27]. Disruptions to school food programs impacted vulnerable populations and food insecure households disproportionately. However, some studies have reported improvements in diet quality and eating habits during the early pandemic [13,28]. In addition, studies have also reported increased sedentary behavior, poorer dietary intakes, and increased alcohol consumption during the COVID-19 pandemic among university students [29].

There has also been a substantial amount of misinformation shared regarding COVID-19, including misinformation related to food and nutrition shared on social media [30-33]. The link between nutrition and immune health is important, and a well-rounded and varied diet supports immune health, but there was an increase in unsubstantiated claims that certain eating patterns, diets, foods, products, or supplements can prevent or cure COVID-19 [31,34-36]. Based on the sheer volume of sudden and novel challenges presented to the public, public health professionals had to quickly adapt services to adhere to social distancing measures and develop strategies to assist the public in dealing with these new challenges, including dealing with misinformation. Van den Broucke [32] suggested that health promotion strategies that promote community engagement can improve health outcomes and strengthen community capacity to address pandemic-related disruptions, and this was supported by others in respect to the COVID-19 pandemic [33,37].

One option that warrants further attention in this situation is social media, as it allows individuals to connect and support one another virtually and facilitate community engagement, and it has been gaining popularity in the dietetic profession and for public health use [38,39]. Social media has also been found to be a cost-effective tool to disseminate health promotion messages across populations that may not be reached using traditional advertising methods, such as minorities or lower socioeconomic groups, and can aid in building a supportive virtual community [40]. In Canada, social media use is very common; a recent report found that 94% of Canadian adults who use the internet have an account on at least one social media platform, and 83%, 51%, and 42% of these individuals had a Facebook, Instagram, and Twitter account in 2020, respectively [41]. This same report also found that in Canada, Instagram had the largest gain among adults who had an account (+14% since 2017) [41]. In addition, during the COVID-19 pandemic, use and analysis of social media has generated substantial interest [42-46]. For example, Pahayahay and Khalili-Mahani [42] investigated the relationship between stress and the media during the first 4 weeks of lockdown due to COVID-19 and found that respondents had a complex relationship with social media. They found that they rely on it for a feeling of social connection and as an information source during isolation. However, they also found that the increased exposure to negative news or misinformation surrounding COVID-19 caused increased stress [42]. Pahayahay and Khalili-Mahani [42] further found that there was a need for positivity in media (eg, news social networks and newsletters) during this time.

Eat Well Saskatchewan (EWS) [47] is a free service that connects residents of the province of Saskatchewan, Canada, to a registered dietitian and provides evidence-based food and nutrition information by phone, email, and social media. EWS noticed an increase in social media use in late March 2020 and, as a result, created evidence-based content related to nutrition and COVID-19 and posted a COVID-19 and nutrition FAQ (frequently asked questions) section on their website [48] to answer questions surrounding immune health, grocery shopping, breastfeeding, and food safety. EWS also began disseminating COVID-19–related social media posts early in the pandemic and saw a 20% spike in performance of posts, suggesting that there was public interest in receiving evidence-based nutrition information from a reliable source. Because of these observations, EWS implemented a positive social media campaign, #eatwellcovid19, that invited residents to share personal stories about how they were eating healthy during the COVID-19 pandemic.

This manuscript seeks to (1) describe the implementation of the #eatwellcovid19 social media campaign and (2) share results from the evaluation of the campaign, which encompassed examining campaign performance using social media metrics and experiences and perspectives of campaign followers using semistructured interviews.

## Methods

### Ethics Approval

This research project was approved by the University of Saskatchewan Behavioural Research Ethics Board (BEH 1975). The analysis of social media analytics received an exemption from the University of Saskatchewan Behavioural Research Ethics Board.

### Setting

Saskatchewan is a multicultural prairie province in Canada that spans over 588,000 square kilometers [49]. The most recent Canadian census was done in 2016 and found that Saskatchewan had a population of 1,098,352, of which 112,490 reported as immigrants; 115,875 reported being a visible minority; and 175,020 reported an Aboriginal identity [49]. Saskatchewan Bureau of Statistics reported that 655,313 residents lived in urban cities; 149,717 lived in towns; 176,535 lived in rural municipalities; 47,308 lived in villages; 56,050 lived on reserve; and 13,429 lived in other areas, such as Crown Colonies or hamlets, in 2016 [50]. The Saskatchewan Bureau of Statistics estimates that as of July 1, 2020, the population of Saskatchewan was 1,178,681 people [51].

### Social Media Campaign Design

This social media campaign, #eatwellcovid19, was led by the registered dietitian at EWS and assisted by the EWS student assistant and researcher, a second-year nutrition student; three nutrition faculty members provided guidance and advice for the campaign on an as-needed basis. EWS can be found on Facebook as Eat Well Saskatchewan, on Instagram as @eatwellsaskatchewan, and on Twitter as @EatWellSask.

In the past, EWS has conducted social media campaigns. One previous campaign (ie, #eatwellchampion) provided a platform for Indigenous people from across Saskatchewan to tell their own stories about the impact that nutrition has had on them. An informal evaluation of social media metrics found that #eatwellchampion posts performed well (eg, commonly shared) and appeared to resonate with followers. The #eatwellcovid19 campaign was partially based on the successful experience of this campaign.

The purpose of the #eatwellcovid19 campaign was to provide a platform for Saskatchewan residents to share their experiences and stories about how they are eating well during the COVID-19 pandemic. EWS announced the #eatwellcovid19 campaign on their social media platforms—Facebook (Facebook, Inc), Instagram (Facebook, Inc), and Twitter (Twitter, Inc)—on April 20, 2020, through a call-out advertisement encouraging residents of Saskatchewan to submit stories on social media platforms (ie, #eatwellcovid19, @eatwellsaskatchewan, and @eatwellsask) or by email; a sample digital call-out poster is presented in Figure S1 in Multimedia Appendix 1. Saskatchewan residents were invited to submit personal stories about how they were eating healthy during the COVID-19 pandemic. They were provided with sample topics and a framework for what to submit. Stories could be submitted using videos, pictures, and/or words. The campaign ran for 16 weeks and, during this time, the EWS team promoted the campaign on their social media platforms through community partners, such as Indigenous Services Canada, and through the University of Saskatchewan online bulletin. Social media advertising was also purchased to promote the campaign beyond the organic reach. Advertisements were created to encourage story submissions from the public and different vulnerable populations. A total of Can $180 was used for paid advertising to encourage story submissions. On Facebook, three posts to encourage story submissions were boosted with advertisements: 12 days for Can $50, 20 days for Can $50, and 20 days for Can $50. In addition, Can $30 was paid to encourage story submissions on Instagram for an unknown duration, and no paid advertisements to encourage story submissions were used on Twitter. In addition, to encourage story submissions, there was a weekly draw for a Can $100 grocery gift card.

To appeal to a variety of followers and address different areas of interest, the campaign included a variety of posts, including stories, supplemental content related to stories, information resources, and winner announcements. Samples of the different types of social media posts are available in Figures S2 to S7 in Multimedia Appendix 1. The EWS team selected story submissions with unique or mass appeal to be developed into feature stories. One to three feature stories were then featured on the EWS social media platforms each week. Supplemental content was curated by the EWS team and was used to complement featured story submissions and provide more information on the subject matter. This included evidence-based nutrition information, links for further reading from reputable sources, recipes, and tips related to stories. For example, if a story sent in by a Saskatchewan resident described eating more berries during the pandemic, nutritional information about berries, recipes, and tips on picking and preparing berries would be shared to complement this story. Of note, no specific criteria were used in choosing the recipes that were shared; a variety of recipes were shared to appeal to the wide demographic of followers. Some of the recipes shared were submitted by campaign followers and others were found and shared by EWS staff. Examples of recipes shared included green pesto rice, watermelon fruit pizza, cattail pollen biscuits, and one for pickled carrots to encourage pickling and to provide education on food preservation. Information resource posts were those that included general COVID-19 nutrition and food information, content shared from other social media pages, and helpful external links not related to any one story. Winner announcements were posts that announced the winner of a gift card for that week. Posters were also used to advertise the campaign and encourage story submissions.

EWS also utilized the 24-hour story feature on Instagram and Facebook to interact with followers and promote the campaign. However, these 24-hour stories should not be confused with the featured story submissions. The content posted using the 24-hour story feature varied and utilized the many interactive features available on the social media applications (eg, quizzes). These features allowed EWS to promote the campaign, conduct polls, provide interactive quizzes, and ask questions to followers.

The EWS team posted #eatwellcovid19 content in a dynamic manner rather than structuring content to be posted at the same time or on a specific day, largely because the content posted was the result of public participation. The EWS team also engaged with EWS social media followers. This engagement was done by promptly responding to and *liking* comments left by followers on posts and responding to and communicating with followers using the social media platforms’ direct messaging features as appropriate (eg, responding to a follower’s private message).

### Social Media Analytics Analysis

Reach, impressions, engagement, and engagement rate regarding the #eatwellcovid19 campaign were determined using Facebook Insights, Instagram Insights, and Twitter Analytics. The definitions of the different social media metrics vary across each social media platform and are provided in Table 1 [52-55].

To begin the social media analytics analysis, the EWS student researcher (JLG) separated #eatwellcovid19 posts from other posts on the EWS pages that were shared during this time. Reach, impressions, and engagements of the #eatwellcovid19 campaign were determined separately for each social media platform, as the metrics provided by each platform are different. Each #eatwellcovid19 post was classified into a category, depending on the type of content shared (eg, *featured story submission* or *campaign poster*). The metrics provided for individual posts were then recorded. Descriptive statistics, specifically the mean and standard deviation of reach, impressions, engagement, and engagement rate for each post category on each platform, were determined using Microsoft Excel (Microsoft Corp). Metrics for Twitter were collected on October 2, 2020, and metrics for Facebook and Instagram were collected on October 5, 2020. Metrics for the 24-hour Instagram and Facebook stories were not captured because of an oversight, as this information disappears soon after the post is made (eg, 28 days on Facebook). Because data collection occurred after this time period, this information was no longer available.

**Table 1:** Definition of key terms for each social media platform.

Key term			Definition
**Facebook**			
	Reach	The number of unique individuals who viewed the listed type of #eatwellcovid19 post [52]	
	Engagement	The number of actions that individuals made with the listed type of #eatwellcovid19 post, including likes, comments, shares, post clicks, etc [53]	
	Impressions	The number of times the listed type of #eatwellcovid19 post entered an individual’s screen (may include more than one view from the same individual) [52]	
**Instagram photos**			
	Reach	The number of individuals who saw the listed type of #eatwellcovid19 post [54]	
	Impressions	The number of times the listed type of #eatwellcovid19 post was seen (may include more than one view from the same individual) [54]	
	Likes	The number of engagements that involved pressing the *like* button on the listed type of #eatwellcovid19 post	
	Comments	The number of engagements that involved leaving a comment on the listed type of #eatwellcovid19 post	
**Instagram videos**			
	Reach	The number of individuals who saw the listed type of #eatwellcovid19 post [54]	
	Likes	The number of engagements that involved pressing the *like* button on the listed type of #eatwellcovid19 video	
	Views	The number of times the listed type of #eatwellcovid19 video was viewed	
**Twitter**			
	Impressions	The number of times the listed type of #eatwellcovid19 post entered a person’s screen (eg, timeline and search results) [55]	
	Engagement	Any interactions on the listed type of #eatwellcovid19 post, including clicks, retweets, replies, likes, etc [55]	
	Engagement rate	This value is calculated by Twitter and is total engagements divided by total impressions for the listed type of #eatwellcovid19 post [55]	

### Semistructured Qualitative Interviews

Reporting of the qualitative interview portion of this manuscript was guided by the Consolidated Criteria for Reporting Qualitative Research (COREQ) checklist [56]. Purposeful sampling [57] was used to select participants to complete a qualitative interview on their experiences and perspectives regarding the #eatwellcovid19 social media campaign. Participants were recruited via the EWS social media pages. A poster advertising the study was posted on the EWS social media pages in August and September 2020. One poster to advertise this study was promoted on Facebook with paid advertising (Can $25 for 5 days). Prospective participants contacted the researcher via telephone or email. After the prospective participant contacted EWS, the researcher screened the individual to determine if they met the study inclusion criteria. Inclusion criteria were as follows: Saskatchewan resident, ≥18 years of age, can speak English, had followed EWS social media accounts, and had the ability to provide informed consent. Once ensuring the participant met the inclusion criteria, the researcher scheduled an interview with the participant via telephone or online.

All telephone or online interviews were conducted remotely by the EWS assistant and student researcher (JLG), a female nutrition student trained in qualitative interview methods. The only individuals present during the interviews were the interviewer and the participants. All interviews occurred in September 2020. There were no established relationships between the interviewer and participants. Prior to beginning the interview, the researcher read the consent form to the participant, which detailed the study objectives and procedures, ensured the participant’s understanding, and obtained oral consent. The interview protocol (Multimedia Appendix 2) contained questions about EWS follower experiences and perspectives regarding the #eatwellcovid19 social media campaign; both clarifying and elaborating probes were used to gather additional information. The interview protocol was developed by all members of the research team and field-tested with one individual to ensure that the questions were appropriate, relevant, and clear and captured the desired information [58]. Interviews were completed until data saturation occurred. Creswell defines data saturation as when “the researcher stops collecting data because fresh data no longer sparks new insights or reveals new properties” [59]. No repeat interviews were conducted. After completion of the interview, participants were mailed a Can $25 grocery store gift card in appreciation of their time. No field notes were taken during the interviews. All interviews were audio recorded and transcribed verbatim by a professional transcriptionist. Transcripts were not returned to participants for comment or corrections. Analysis of the interview transcripts was conducted using conventional content analysis [60]. All interview transcripts were coded inductively by the student researcher (JLG) using NVivo 12 (QSR International). The coding process allowed inductive categories and themes to emerge from the data [60]; no a priori codes were used. All interview transcripts, coding, and the draft themes were reviewed by a second member of the research team (JRLL) with experience in qualitative research. Discrepancies were discussed and consensus was reached.

## Results

### Overview

The #eatwellcovid19 campaign received 75 story submissions from 74 Saskatchewan residents, and 42 of these stories were featured on EWS social media pages. The main themes seen in the submitted stories about how Saskatchewan residents were eating healthy during the COVID-19 pandemic can be found in Table 2. The top three themes of submitted stores were increased time spent cooking or trying new recipes, grocery shopping habits, and gardening. Of note, 2 featured stories appeared in local Saskatchewan newspapers and the top featured story differed by platform.

**Table 2:** Themes of submitted stories for the #eatwellcovid19 social media campaign.

Story theme	Stories submitted (N=75), n (%)	Examples
Increased time spent cooking or trying new recipes	21 (28)	Budget-friendly recipes, trying different cuisines, bread baking, and improved nutrition from more homemade meals
Grocery shopping habits	17 (23)	Stocking up on nonperishable items, change in frequency of grocery shopping trips, feelings of unease in the grocery store, and individual vs family outing
Gardening	12 (16)	Tried gardening for the first time, expanded garden, and utilized a community garden
Traditional skills	8 (11)	Foraging, hunting, and preserving
Increased family time and family meals	6 (8)	Change in mealtime patterns, more time to eat together, and involving kids in the kitchen
Other	11 (15)	Food waste and pregnancy

### Social Media Analytics

EWS’s Facebook page began the campaign at 1524 page likes and ended the campaign with 1780 page likes, representing 14% growth. A notable outcome for EWS’s Instagram page was the 30% growth in followers from the beginning of the campaign. The EWS Instagram account began the campaign with 262 followers and ended the campaign with 376 followers. The EWS Twitter account had 132 followers at the beginning of the campaign and ended the campaign with 150 followers, representing 12% growth. Analyses of social media metrics for the different types of posts are shown in Tables 3-6.

The focus of the #eatwellcovid19 campaign was on Facebook, as this was where EWS had the largest social media following. In total, 100 #eatwellcovid19 posts, not including 24-hour stories, were shared on the EWS Facebook page and, overall, there were 9575 engagements with this content on Facebook. On Facebook, after excluding post types that were sometimes promoted with paid advertising, featured story posts (n=38) had the highest mean engagement (133 engagements/post). The mean engagement of these posts was more than three times higher than the mean engagement of supplemental content related to featured story posts (40 engagements/post), more than four times higher than the mean engagement for winner announcement posts (30 engagements/post), and more than six times higher than the mean engagement for information resource posts (20 engagements/post). The campaign was also seen by many individuals on Facebook. In total, the #eatwellcovid19 campaign had a reach of 100,571 and left 128,818 impressions. Similar to engagement, for post types that were not promoted with paid advertising, featured story submissions had higher reach and impressions compared to other types of posts.

**Table 3:** Metrics of the #eatwellcovid19 social media posts on Facebook.

Facebook post category	Posts or stories, n	Engagement, mean (SD)	Reach, mean (SD)	Impressions, mean (SD)
Featured story submission	38	133 (212)	824 (773)	987 (959)
Supplemental content related to featured story	24	40 (46)	576 (684)	673 (767)
Campaign poster^a^	8	361 (487)	5433 (5353)	7679 (7340)
Information resource	23	20 (13)	360 (84)	410 (95)
Winner announcement	7	30 (17)	526 (224)	612 (225)
24-hour story	27^b^	N/A^c^	N/A	N/A

^a^Some campaign posters on Facebook were boosted with paid advertising.

^b^This number is approximate.

^c^N/A: not applicable; this information was unable to be collected from Facebook.

**Table 4:** Metrics of the #eatwellcovid19 social media posts on Instagram.

Instagram post category	Posts or stories, n	Likes, mean (SD)	Comments, mean (SD)	Reach, mean (SD)	Impressions, mean (SD)
Featured story submission	36	15 (8)	0.4 (0.8)	171 (38)	214 (52)
Supplemental content related to featured story	5	11 (5)	0.2 (0.4)	160 (16)	183 (21)
Campaign poster^a^	5	23 (30)	0 (0)	584 (959)	850 (1494)
Information resource	2	10 (7)	0 (0)	153 (28)	177 (42)
Winner announcement	5	12 (6)	0.2 (0.4)	160 (27)	187 (39)
24-hour story	58	N/A^b^	N/A	N/A	N/A

^a^Some campaign posters on Instagram were boosted with paid advertising.

^b^N/A: not applicable; this information was unable to be collected from Instagram.

**Table 5:** Metrics of the #eatwellcovid19 social media videos on Instagram.

Instagram video category	Posts, n	Likes, mean (SD)	Reach, mean (SD)	Views, mean (SD)
Featured story submission	2	14 (3)	155 (12)	103 (27)

**Table 6:** Metrics of the #eatwellcovid19 social media posts on Twitter.

Twitter post category	Posts, n	Engagements, mean (SD)	Engagement rate^a^, mean (SD)	Impressions, mean (SD)
Featured story submission	26	10 (10)	3 (2)	281 (212)
Supplemental content related to featured story	3	10 (10)	3 (0.6)	339 (368)
Campaign poster	7	18 (18)	2 (1)	890 (830)
Information resource	1	1 (0)	1 (0)	218 (0)
Winner announcement	2	4 (2)	2 (1)	217 (0.7)

^a^This value is calculated by Twitter and is total engagements divided by total impressions for the listed type of #eatwellcovid19 post [55].

Results on Instagram campaign performance using social media metrics were similar to Facebook. In total, 55 #eatwellcovid19 posts, not including 24-hour stories, were shared on EWS’s Instagram page and these posts received a total of 823 likes and 15 comments. After excluding post types that were sometimes promoted with paid advertising, featured story posts (n=36) had higher numbers of mean likes (15 likes/post) and mean comments (0.4 comments/post) compared to other types of posts, such as winner announcements (mean 12 likes/post and mean 0.2 comments/post), supplemental content related to the featured story (mean 11 likes/post and mean 0.2 comments/post), and information resource posts (mean 10 likes/post and mean 0 comments/post). The campaign was also seen by many followers on Instagram. In total, on Instagram, the #eatwellcovid19 campaign had a reach of 11,310 and left 14,145 impressions. Similar to engagement, for post types that were not promoted with paid advertising, featured story submissions had higher reach and more impressions compared to other types of posts.

Results on Twitter campaign performance using social media metrics were different compared to Facebook and Instagram. In total, 39 #eatwellcovid19 posts were shared on the EWS Twitter page and these posts received 424 engagements. Regarding engagement, the campaign posters received the most engagements (mean 18 engagements/post), and this was followed by featured story submissions (mean 10 engagements/post), supplemental content related to featured story posts (mean 10 engagements/post), winner announcements (mean 4 engagements/post), and information resource posts (mean 1 engagement/post). The campaign was also seen by many followers on Twitter. In total, there were 15,199 impressions, averaging an engagement rate of 3%. Impressions were highest for campaign posters, followed by supplemental content related to featured story posts, featured story posts, information resource posts, and winner announcement posts.

### Semistructured Qualitative Interviews

In total, 86 people indicated interest in participating in a qualitative interview, 35 people responded to the initial screening to proceed further, 12 were ineligible based on pre-established criteria, and 20 interviews were conducted. No participants dropped out of the study after the interviews took place. The average interview took 8 minutes and 54 seconds (range 4 minutes and 39 seconds to 12 minutes and 31 seconds). Participant demographics are listed in Table 7.

**Table 7:** Demographics of interview participants.

Demographic		Participants (N=20), n (%)
**Age category (years)**		
	18-30	8 (40)
	31-50	10 (50)
	51-70	2 (10)
**Gender**		
	Female	18 (90)
	Male	2 (10)
	Other	0 (0)
	Prefer not to say	0 (0)
**Place of residence**		
	Urban	17 (85)
	Rural	3 (15)
	Remote	0 (0)
**Immigrant status**		
	Canadian citizen or nonrecent immigrant (>5 years residing in Canada)	19 (95)
	Recent immigrant (<5 years residing in Canada)	1 (5)

The participants followed EWS for variable amounts of time; of note, most of these participants followed EWS on Facebook and Instagram, and only one participant followed EWS on Twitter. Analysis of the interview transcripts showed that followers appreciated the content shared during the #eatwellcovid19 campaign. There appeared to be two types of campaign followers. The first type of follower valued the positive personal stories shared in a time with increased negative news, the stories resonated with them, and they felt a connection to their community by following this campaign. A notable quote from an interviewee demonstrated that the storytelling format provided a connection to the community in a time where physical connection was not possible:

I think that there’s a lot of value in taking people’s stories, lived experiences, and sharing it with others because I find, a lot of the times, we are living in silos and we’re all thinking, “I’m the only one who struggles with food insecurity. I’m the only one that can’t figure out how to make healthy meals for my kids that they’ll actually eat,” and really that’s not the case at all, but with the opportunity that comes with working with other people that says, “Me too. I struggle with this as well.” It brings connection and community within these silos we have and just makes us know that we’re not alone in our struggles. Everybody has concerns about food and how to be healthy.

Participant #13, female, 18 to 30 years of age

The second type of follower was content driven; these followers appreciated the evidence-based information given and followed the campaign for recipes, meal planning tips, and food safety information during the COVID-19 pandemic. A notable quote from an interviewee demonstrated that the campaign helped them to overcome grocery shopping challenges during the COVID-19 pandemic:

There was this link to “How to grocery shop and prioritize during COVID.” And there was lots of information about, “Don’t over-buy. Make sure you have this on stock and this on stock,” and it’s really good for me because I’m kind of not the great—I don’t have too much income, and so I have to prioritize and budget pretty well. So seeing a list like that was pretty good for helping me to try to prioritize what I need during this time.

Participant #14, female, 18 to 30 years of age

Both types of followers appreciated and emphasized the importance of sharing local, Saskatchewan-based content to meet the unique needs of Saskatchewan residents.

## Discussion

### Principal Findings

Overall, numerous stories on various topics related to healthy eating were submitted to the #eatwellcovid19 social media campaign. In general, the social media metrics revealed that featured stories had better engagement compared to other types of posts (eg, supplemental content related to featured stories). The #eatwellcovid19 campaign also reached many individuals and made numerous impressions on social media. The semistructured interviews revealed that there were two types of campaign followers: those who appreciated reading the featured story posts, as they found that these posts helped them to feel connected to their community, and those who appreciated the evidence-based information. The lessons learned from #eatwellcovid19 are helpful to those looking to develop their own social media campaigns for health purposes.

In total, EWS received 75 story submissions from 74 individuals for #eatwellcovid19 over the 16-week campaign, which was substantial. The topics of the submitted stories were broad and included, for example, gardening, traditional skills, grocery shopping, family time, and meals. Because of the broad nature of story topics, they seemed to appeal to a wide variety of followers. This campaign was also unique, as much of the campaign content was organic and provided by the social media followers rather than developed by researchers or health professionals, which is more typical in social media campaigns [61]. We also found that Can $100 grocery gift card draws were likely a good incentive for the public to submit their personal stories. This finding is not surprising, as Perrault et al also found in their survey study on college health social media pages that incentives were desired by students to follow, interact with content, and share content with others [62]. Of note, a few of the gift card winners commented on how helpful the Can $100 grocery gift card was going to be in the economically challenging times.

In terms of engagement on Facebook, and likes and comments on Instagram, featured story submissions appeared to perform the strongest relative to other types of campaign posts, including supplemental content related to the featured story, winner announcements, and information resources. This finding is similar to a study by Gabarron et al who found that on the Facebook, Instagram, and Twitter pages for the Norwegian Diabetes Association, posts containing stories and personal interviews had the most likes, comments, and shares compared to other types of posts over a 3-year period [63]. They also found that posts with information on recipes and food-related information received less engagement [63]. In addition, Pedersen et al also found that in a social media campaign on human papillomavirus vaccine promotion, posts with personal stories had the best engagement and created positive comments [64]. These findings were also not surprising, as Heldman et al have suggested that soliciting user-generated content is something that should be encouraged to promote user engagement when using social media for public health purposes [39]. This suggests that use of personal stories and experiences is a valuable way to promote engagement in social media for health purposes.

Although featured story submissions had the highest engagement on Facebook and Instagram, using likes and comments, this was not the case with Twitter. On Twitter, the posters advertising the campaign had the highest engagement, and featured stories and supplemental content related to the featured story had similar engagement levels. These findings may be attributed to the 280-character limit imposed by Twitter. As a result of this character limit, the EWS team had to shorten and paraphrase the stories to fit in the allotted space. This paraphrasing often removed the bulk of the story and, as a result, may have diminished the personal connection that followers found in the featured story submissions on the other platforms. Another possible reason for this finding is that Facebook, Instagram, and Twitter are unique platforms, and each platform has a different user demographic [41]. For example, Instagram has a large number of users aged 18 to 24 years [41]. In addition, in a study examining preferences for a diabetes health promotion campaign, Gabarron et al [65] determined that Facebook was the most preferred platform overall for health promotion campaigns, and Twitter was the least preferred. These preferences could also explain different findings in the relative performance of different types of posts on the different platforms. It is important when developing and implementing social media campaigns for health-related purposes that the developers investigate and determine which platform is most appropriate to choose, as preferences change frequently and new platforms are always emerging [66]. Pilot studies and survey research of target followers may be a strategy to gather this information.

Overall, followers appreciated the positive messages and content of this campaign, especially in a time of increased exposure to negative news. This finding aligns with results found by Pahayahay and Khalili-Mahani [42] described earlier. Additionally, there is a growing concern surrounding the quality of information shared on social media, including the presence of nonevidence-based health information; therefore, recommendations have been made for health professionals to counteract this misinformation by providing simple, evidence-based content through social media channels [67]. Interview participants felt that the #eatwellcovid19 campaign shared valuable stories and reliable and attainable nutrition information and advice. It was reassuring for followers that content was evidence based, positive, and vetted by a dietitian. Different kinds of followers followed the campaign, so a variety of content is very important to appeal to everyone. Although some followers liked how the campaign helped to connect individuals when they were apart from others, others still followed the campaign for simple food and nutrition information, and not just for community connection. This should be taken into consideration when developing future health promotion campaigns.

EWS is a relatively new service in Saskatchewan, established in March 2019, and has since been actively growing its online social media following. As EWS’s following continues to grow, the performance of future campaigns will likely improve, especially considering that 158,162 impressions were made on a combined total of only 2306 followers across the three social media platforms. Focusing on the social media metrics of the campaign, Facebook performed the best, which is not surprising because it is also EWS’s most followed platform. The campaign made more impressions on Twitter than it did on Instagram, which is surprising because EWS has more followers on Instagram than Twitter and 19 more #eatwellcovid19 posts were shared on Instagram compared to Twitter. All platforms experienced a growth in their following, with 30% growth in EWS Instagram’s following being the most substantial. The growth in followers across platforms because of the #eatwellcovid19 campaign suggests that health professionals and health organizations with a small social media following could use a health promotion social media campaign to establish themselves on social media and increase their following [62].

The design of our campaign could be viewed as a strength or a limitation, as the success of the campaign was dependent on the participation of EWS social media followers and their willingness to submit stories. Due to the high volume and quality of the submissions received, the #eatwellcovid19 campaign was able to share numerous stories on a variety of topics as well as credible nutrition information that supplemented those stories. However, if EWS had only received a small number of stories for the campaign, there would not have been enough material to make it successful. Although personal stories are empowering and are a valuable tool for public engagement, it can be time-consuming to collect stories and turn them into posts, and it is possible that the content submitted by followers may not align well with the overall goals or message of the campaign. Therefore, health professionals who have specific content to share in a timely manner may have a difficult time incorporating user-shared stories into their campaign design.

A limitation of the #eatwellcovid19 evaluation was that we were unable to determine if eating behavior changes occurred as a direct result of the campaign; however, some qualitative data suggest that small behavior changes may have occurred or been prompted by the campaign. Another limitation of the study was that the interviewer for the semistructured qualitative interviews was not external to the campaign. In addition, we are unsure of the demographics of EWS followers, as social media metrics from each platform do not provide detailed follower demographic information (ie, where a follower lives). Therefore, we are also unsure if the participants who completed interviews are directly representative of campaign followers. A final limitation and a lesson learned for future social media campaigns is that detailed 24-hour story metrics are only available for a short time on Facebook and Instagram (eg, 28 days on Facebook). We only learned after the campaign was finished that Instagram and Facebook 24-hour story metrics could not be collected, as we collected our data past the time point of where this information was available. As a result, we were only able to capture the number of stories shared. This oversight was unfortunate because there was a substantial amount of reach and follower engagement that occurred with using the 24-hour stories in the #eatwellcovid19 campaign. In future campaigns, it would be valuable to record the number of stories shared and continually collect these metrics, such as reach and engagement, to better evaluate campaign performance. In addition, use of the 24-hour story feature in social media is a promising area of future research for health-related social media campaigns.

### Conclusions

Numerous stories were shared to #eatwellcovid19 that showcased various strategies on how individuals were eating well during the COVID-19 pandemic. On Instagram and Facebook, posts that featured these stories had the highest engagement compared to other post types (eg, supplemental content related to the feature story). During this campaign, EWS’s social media following increased by more than 10% on each platform. The #eatwellcovid19 campaign can be used as a reference for other health care professionals interested in the design of future social media campaigns.

## Appendix

Multimedia Appendix 1 Examples of social media content used in #eatwellcovid19.

Multimedia Appendix 2 Interview protocol.

## Abbreviations

COREQ: Consolidated Criteria for Reporting Qualitative Research
EWS: Eat Well Saskatchewan
FAQ: frequently asked questions

## References

[1] (2020). Listings of WHO's response to COVID-19. World Health Organization.

[2] (2020). Coronavirus disease (COVID-19). World Health Organization.

[3] (2020). Modes of transmission of virus causing COVID-19: Implications for IPC precaution recommendations. World Health Organization.

[4] Coronavirus disease (COVID-19): Prevention and risks. Government of Canada.

[5] Individual and community-based measures to mitigate the spread of COVID-19 in Canada. Government of Canada.

[6] Shahab S (2021). Public Health Order (Under Subsection 45(2) of The Public Health Act, 1994 and section 25.2(2) of The Disease Control Regulations) Control of Transmission of 2019 Novel Coronavirus.

[7] Shahab S (2020). Public Health Order (Under Subsection 38 and Subsection 45(2) of The Public Health Act, 1994) Control of Transmission of 2019 Novel Coronavirus.

[8] Schmidt C, Goetz S, Rocker S, Tian Z (2020). Google searches reveal changing consumer food sourcing in the COVID-19 pandemic. J Agric Food Syst Community Dev.

[9] (2020). Canadian consumers prepare for COVID-19. Statistics Canada.

[10] Martin-Neuninger R, Ruby M (2020). What does food retail research tell us about the implications of coronavirus (COVID-19) for grocery purchasing habits?. Front Psychol.

[11] Deaton BJ, Deaton BJ (2020). Food security and Canada's agricultural system challenged by COVID‐19. Can J Agric Econ.

[12] Wolfson JA, Leung CW (2020). Food insecurity and COVID-19: Disparities in early effects for US adults. Nutrients.

[13] Ben Hassen T, El Bilali H, Allahyari MS, Berjan S, Fotina O (2021). Food purchase and eating behavior during the COVID-19 pandemic: A cross-sectional survey of Russian adults. Appetite.

[14] Sarney G (2016). CFDR study reveals consumer confusion related to nutrition information. Canadian Foundation for Dietetic Research.

[15] (2015). Tracking nutrition trends 2015. Canadian Foundation for Dietetic Research.

[16] Ali SH, Imbruce VM, Russo RG, Kaplan S, Stevenson K, Mezzacca TA, Foster V, Radee A, Chong S, Tsui F, Kranick J, Yi SS (2021). Evaluating closures of fresh fruit and vegetable vendors during the COVID-19 pandemic: Methodology and preliminary results using omnidirectional street view imagery. JMIR Form Res.

[17] Richards TJ, Rickard B (2020). COVID‐19 impact on fruit and vegetable markets. Can J Agric Econ.

[18] Anelich LECM, Lues R, Farber JM, Parreira VR (2020). SARS-CoV-2 and risk to food safety. Front Nutr.

[19] Han J, Zhang X, He S, Jia P (2021). Can the coronavirus disease be transmitted from food? A review of evidence, risks, policies and knowledge gaps. Environ Chem Lett.

[20] (2020). COVID-19 and food safety: Guidance for food businesses. Interim guidance. World Health Organization.

[21] Kumar S, Singh R, Kumari N, Karmakar S, Behera M, Siddiqui AJ, Rajput VD, Minkina T, Bauddh K, Kumar N (2021). Current understanding of the influence of environmental factors on SARS-CoV-2 transmission, persistence, and infectivity. Environ Sci Pollut Res Int.

[22] Adams EL, Caccavale LJ, Smith D, Bean MK (2020). Food insecurity, the home food environment, and parent feeding practices in the era of COVID-19. Obesity (Silver Spring).

[23] Men F, Tarasuk V (2021). Food insecurity amid the COVID-19 pandemic: Food charity, government assistance, and employment. Can Public Policy.

[24] Dondi A, Candela E, Morigi F, Lenzi J, Pierantoni L, Lanari M (2020). Parents' perception of food insecurity and of its effects on their children in Italy six months after the COVID-19 pandemic outbreak. Nutrients.

[25] Niles MT, Bertmann F, Belarmino EH, Wentworth T, Biehl E, Neff R (2020). The early food insecurity impacts of COVID-19. Nutrients.

[26] Carroll N, Sadowski A, Laila A, Hruska V, Nixon M, Ma DW, Haines J, on behalf of the Guelph Family Health Study (2020). The impact of COVID-19 on health behavior, stress, financial and food security among middle to high income Canadian families with young children. Nutrients.

[27] Noyes I, Lyle N (2021). COVID-19 and school food: The impact of the early stages of the coronavirus pandemic on student nutrition programs in Ontario. J Agric Food Syst Community Dev.

[28] Lamarche B, Brassard D, Lapointe A, Laramée C, Kearney M, Côté M, Bélanger-Gravel A, Desroches S, Lemieux S, Plante C (2021). Changes in diet quality and food security among adults during the COVID-19-related early lockdown: Results from NutriQuébec. Am J Clin Nutr.

[29] Bertrand L, Shaw KA, Ko J, Deprez D, Chilibeck PD, Zello GA (2021). The impact of the coronavirus disease 2019 (COVID-19) pandemic on university students' dietary intake, physical activity, and sedentary behaviour. Appl Physiol Nutr Metab.

[30] Lee JJ, Kang K, Wang MP, Zhao SZ, Wong JYH, O'Connor S, Yang SC, Shin S (2020). Associations between COVID-19 misinformation exposure and belief with COVID-19 knowledge and preventive behaviors: Cross-sectional online study. J Med Internet Res.

[31] van der Linden S, Roozenbeek J, Compton J (2020). Inoculating against fake news about COVID-19. Front Psychol.

[32] Van den Broucke S (2020). Why health promotion matters to the COVID-19 pandemic, and vice versa. Health Promot Int.

[33] Adams KK, Baker WL, Sobieraj DM (2020). Myth busters: Dietary supplements and COVID-19. Ann Pharmacother.

[34] Health product advertising incidents related to COVID-19. Government of Canada.

[35] Gleeson M, Nieman DC, Pedersen BK (2004). Exercise, nutrition and immune function. J Sports Sci.

[36] Henrina J, Lim MA, Pranata R (2021). COVID-19 and misinformation: How an infodemic fuelled the prominence of vitamin D. Br J Nutr.

[37] Chong YY, Cheng HY, Chan HYL, Chien WT, Wong SYS (2020). COVID-19 pandemic, infodemic and the role of eHealth literacy. Int J Nurs Stud.

[38] Dumas A, Lapointe A, Desroches S (2018). Users, uses, and effects of social media in dietetic practice: Scoping review of the quantitative and qualitative evidence. J Med Internet Res.

[39] Heldman AB, Schindelar J, Weaver JB (2013). Social media engagement and public health communication: Implications for public health organizations being truly “social”. Public Health Rev.

[40] Moorhead SA, Hazlett DE, Harrison L, Carroll JK, Irwin A, Hoving C (2013). A new dimension of health care: Systematic review of the uses, benefits, and limitations of social media for health communication. J Med Internet Res.

[41] Gruzd A, Mai P (2020). The state of social media in Canada 2020. SSRN.

[42] Pahayahay A, Khalili-Mahani N (2020). What media helps, what media hurts: A mixed methods survey study of coping with COVID-19 using the media repertoire framework and the appraisal theory of stress. J Med Internet Res.

[43] Graffigna G, Bosio C, Savarese M, Barello M, Barello S (2020). "#I-Am-Engaged": Conceptualization and first implementation of a multi-actor participatory, co-designed social media campaign to raise Italians citizens' engagement in preventing the spread of COVID-19 virus. Front Psychol.

[44] Lucini D, Gandolfi C, Antonucci C, Cavagna A, Valzano E, Botta E, Chiari M, Mameli L, Nahum M, Brambilla MM, Castaldi S, Biganzoli E (2020). #StayHomeStayFit: UNIMI's approach to online healthy lifestyle promotion during the COVID-19 pandemic. Acta Biomed.

[45] Saleh SN, Lehmann CU, McDonald SA, Basit MA, Medford RJ (2021). Understanding public perception of coronavirus disease 2019 (COVID-19) social distancing on Twitter. Infect Control Hosp Epidemiol.

[46] Tsao S, Chen H, Tisseverasinghe T, Yang Y, Li L, Butt ZA (2021). What social media told us in the time of COVID-19: A scoping review. Lancet Digit Health.

[47] Eat Well Saskatchewan.

[48] COVID-19 FAQ. Eat Well Saskatchewan.

[49] Census Profile, 2016 Census: Saskatchewan [Province] and Canada [Country]. Statistics Canada.

[50] (2017). Saskatchewan Population Report: 2016 Census of Canada.

[51] (2020). Saskatchewan Quarterly Population Report: Second Quarter 2020.

[52] What's the difference between Page views, reach and impressions on Facebook?. Facebook Help Center.

[53] Post engagement. Facebook for Business: Business Help Center.

[54] Instagram post reach. Facebook for Business: Business Help Center.

[55] Tweet and video activity dashboards. Twitter Ads Help Center.

[56] Tong A, Sainsbury P, Craig J (2007). Consolidated criteria for reporting qualitative research (COREQ): A 32-item checklist for interviews and focus groups. Int J Qual Health Care.

[57] Patton MQ (2002). Qualitative Research and Evaluation Methods. 3rd edition.

[58] Kallio H, Pietilä AM, Johnson M, Kangasniemi M (2016). Systematic methodological review: Developing a framework for a qualitative semi-structured interview guide. J Adv Nurs.

[59] Creswell JW (2014). Glossary. Research Design: Qualitative, Quantitative, and Mixed Methods Approaches. 4th edition.

[60] Hsieh H, Shannon SE (2005). Three approaches to qualitative content analysis. Qual Health Res.

[61] Graham JE, Moore JL, Bell RC, Miller T (2019). Digital marketing to promote healthy weight gain among pregnant women in Alberta: An implementation study. J Med Internet Res.

[62] Perrault EK, Hildenbrand GM, McCullock SP, Schmitz KJ, Dolick KN (2019). Hashtag health: College health on social media and students' motivations to follow, interact, and share their social media content. Health Promot Pract.

[63] Gabarron E, Larbi D, Dorronzoro E, Hasvold P, Wynn R, Årsand E (2020). Factors engaging users of diabetes social media channels on Facebook, Twitter, and Instagram: Observational study. J Med Internet Res.

[64] Pedersen EA, Loft LH, Jacobsen SU, Søborg B, Bigaard J (2020). Strategic health communication on social media: Insights from a Danish social media campaign to address HPV vaccination hesitancy. Vaccine.

[65] Gabarron E, Dorronzoro E, Bradway M, Rivera-Romero O, Wynn R, Årsand E (2018). Preferences and interests of diabetes social media users regarding a health-promotion intervention. Patient Prefer Adherence.

[66] Ortiz-Ospina E (2019). The rise of social media. Our World in Data.

[67] Chou WS, Oh A, Klein WMP (2018). Addressing health-related misinformation on social media. JAMA.

