# Supplementary material for: Evaluation of a Social Media Campaign in Saskatchewan to Promote Healthy Eating During the COVID-19 Pandemic: Social Media Analysis and Qualitative Interview Study
Source: J Med Internet Res. 2021 Jul 21;23(7):e27448. doi: 10.2196/27448 (PMC8297600; doi:10.2196/27448)

Multimedia Appendix 1

Figure S1: Example of a campaign poster used to encourage personal story submissions.

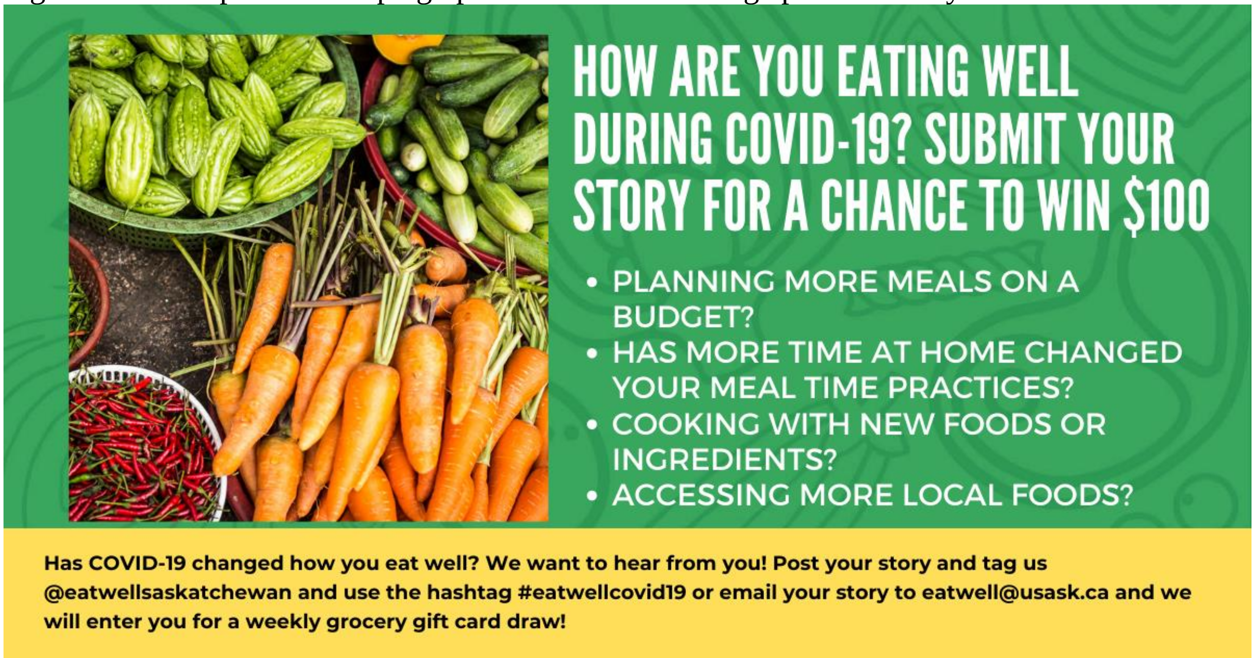

Figure S2, Figure S3: Examples of 24-hour story to encourage follower engagement and story submission.

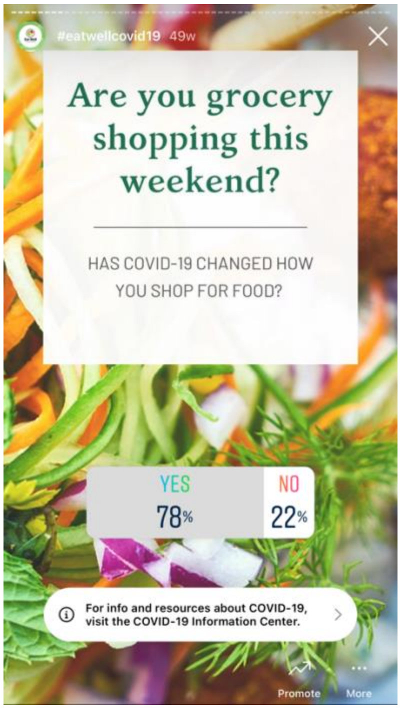

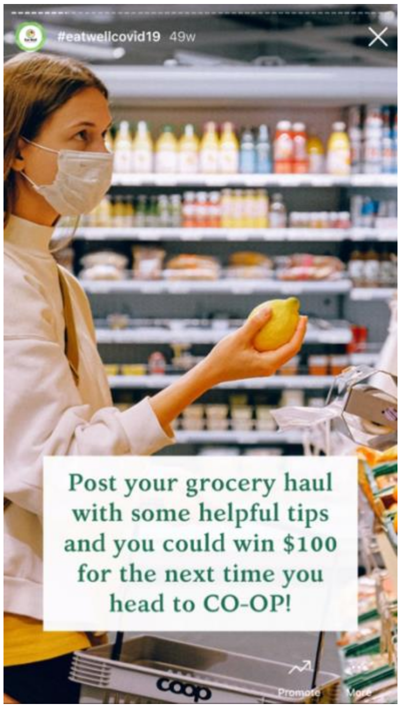

Figure S4: Example of a personal story shared. This story was shared with no caption.

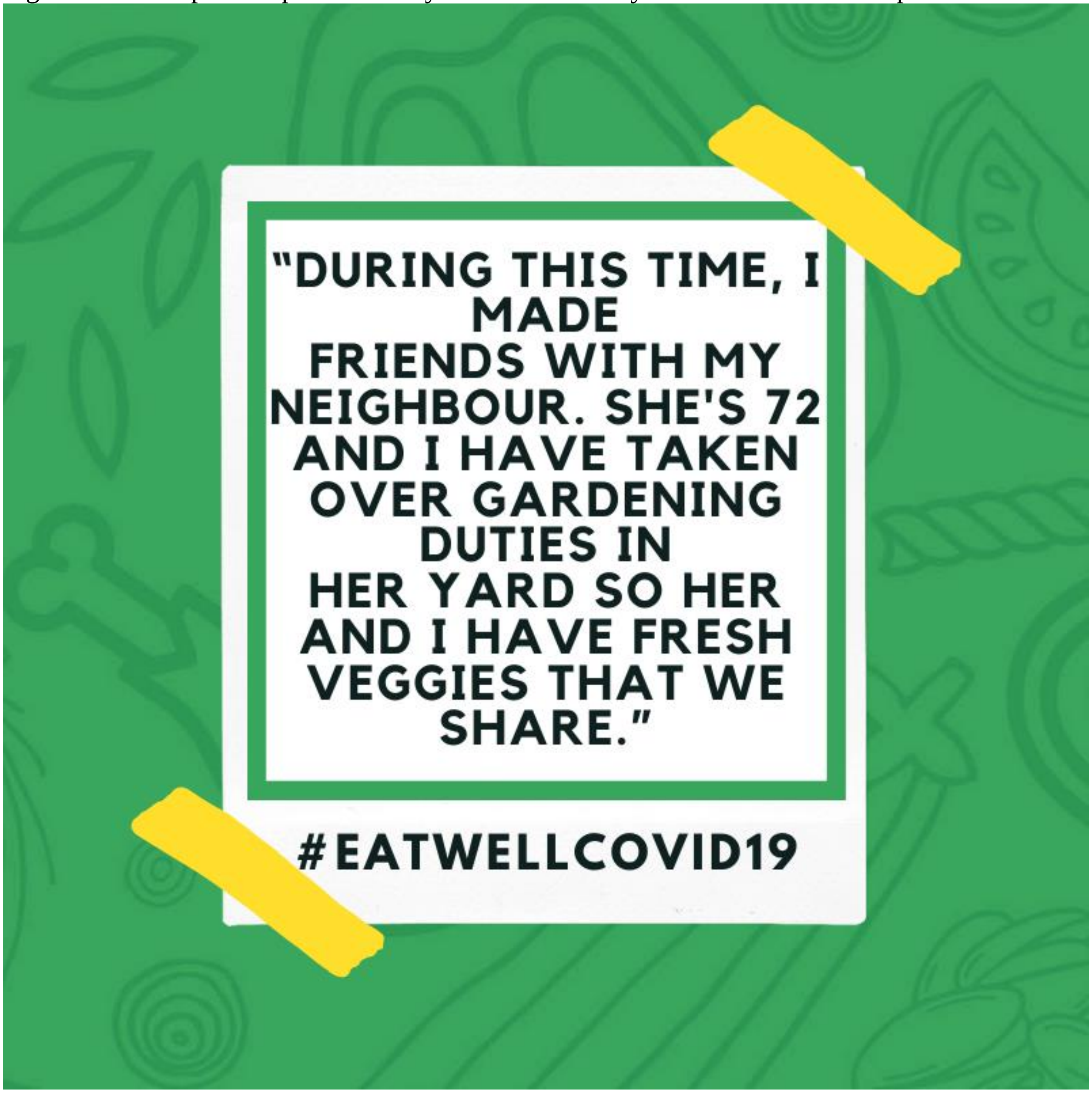

Figure S5, Figure S6, Figure S7: Example of a featured submission story. Name has been redacted and permission was granted for sharing of personal photos.

This was captioned, “Have you been gardening during COVID-19? Saskatchewan resident, (*name of person*), has started a vegetable garden and is enjoying her time outdoors to absorb her daily dose of vitamin D. Check out her story below.

Have you been growing your own food during COVID-19? Tell us about it! Send us your story to eatwell@usask.ca or tag us @eatwellsaskatchewan and use #eatwellcovid19.”

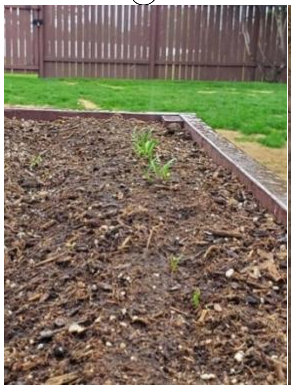

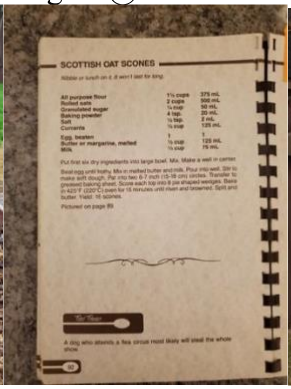

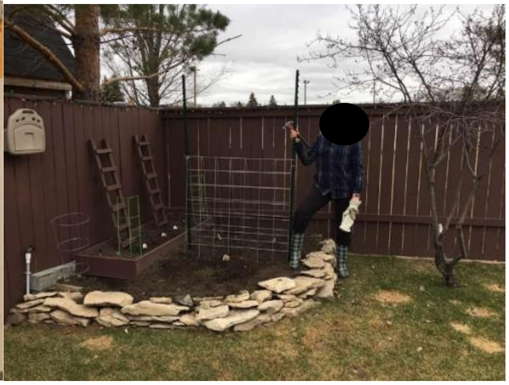

Supplement: Multimedia Appendix 1 [file jmir_v23i7e27448_app1.pdf]
