# Supplementary material for: Evaluation of a Social Media Campaign in Saskatchewan to Promote Healthy Eating During the COVID-19 Pandemic: Social Media Analysis and Qualitative Interview Study
Source: J Med Internet Res. 2021 Jul 21;23(7):e27448. doi: 10.2196/27448 (PMC8297600; doi:10.2196/27448)
Supplement: Multimedia Appendix 2 [file jmir_v23i7e27448_app2.pdf]

Multimedia Appendix 2

Interview Questions

1) How long have you been following Eat Well Saskatchewan's social media pages? Which pages do you follow?
2) How often do you view content on these pages? What is it that interests you most? Why?
3) What are your reasons for following the Eat Well Saskatchewan social media pages?
4) What are your thoughts on the stories and educational content related to COVID-19 posted on the Eat Well Saskatchewan social media pages? (Follow-up question: What did you find most useful? Videos, website links, recipes, images, stories?)
5) Can you tell us something that you learned during this campaign?
6) What was your favourite #eatwellcovid19 story submission or post?
7) Did you send in a COVID-19 story to the campaign? Why or why not?
8) Have you made any changes as a result of this campaign that you believe helped you and your family cope with COVID-19? If yes, are you willing to share?
9) Should a similar campaign be run in the future on a related topic, would you participate? Why or why not?
10) Is there anything else that you would like to add?

What is your age category? 18-30 years, 31-50 years, 51-70 years, 71+ years
What is your gender? Male, Female, Other, Prefer not to say
Where do you live in Saskatchewan? Urban, Rural, Northern/remote community (please explain)
Are you new to Canada within the last 5 years?
Thank you for participating in this interview. Before we end the interview, is there anything else you would like to share?
